# Job stress and work from home during the COVID-19 pandemic among Japanese workers: a prospective cohort study

**DOI:** 10.1080/21642850.2022.2163248

**Published:** 2023-01-20

**Authors:** Kazunori Ikegami, Hajime Ando, Kosuke Mafune, Mayumi Tsuji, Seiichiro Tateishi, Kiminori Odagami, Keiji Muramatsu, Yoshihisa Fujino, Akira Ogami

**Affiliations:** a Department of Work Systems and Health, Institute of Industrial Ecological Sciences, University of Occupational and Environmental Health, Kitakyusyu, Japan; b Department of Mental Health, Institute of Industrial Ecological Sciences, University of Occupational and Environmental Health, Kitakyusyu, Japan; c Department of Environmental Health, School of Medicine, University of Occupational and Environmental Health, Kitakyusyu, Japan; d Disaster Occupational Health Center, Institute of Industrial Ecological Sciences, University of Occupational and Environmental Health, Kitakyusyu, Japan; e Department of Occupational Health Practice and Management, Institute of Industrial Ecological Sciences, University of Occupational and Environmental Health, Kitakyusyu, Japan; f Department of Public Health, School of Medicine, University of Occupational and Environmental Health, Kitakyusyu, Japan; g Department of Environmental Epidemiology, Institute of Industrial Ecological Sciences, University of Occupational and Environmental Health, Kitakyusyu, Japan

**Keywords:** Work from home, office worker, job stress, Brief Job Stress Questionnaire, COVID-19

## Abstract

**Objectives:**

The work style reforms and the coronavirus disease pandemic in Japan have prompted efforts toward teleworking, mainly work from home (WFH). This study aimed to prospectively evaluate the impact of WFH on job stress among Japanese workers.

**Methods:**

This online survey-based prospective cohort study was conducted from December 2020 (baseline) to December 2021 (1-year follow-up) using self-administered questionnaires. At baseline, 27,036 participants completed the questionnaires, whereas 18,560 (68.7%) participated in the 1-year follow-up. After excluding the 11,604 participants who left or changed workplaces within 1 year or who were physical laborers and hospitality workers, data from 6,956 participants were analyzed. We asked participants about WFH frequency at baseline and conducted a follow-up using the Brief Job Stress Questionnaire (BJSQ). Participants were divided into four groups according to WFH frequency. The odds ratios of poor states of the association of the four subscales (job demand, job control, supervisor support, and coworker support) determined by the BJSQ with WFH frequency were estimated using a multilevel logistic model.

**Results:**

In both the gender–age adjusted and multivariate models, compared to the non-WFH group, the medium and low WFH groups were less likely to have poor job control, whereas the high WFH group were likely to have similar levels as the non-WFH group. In both models, compared to non-WFH participants, the high WFH group were more likely to have poor supervisor and coworker support.

**Conclusions:**

High-frequency WFH requires further attention, because it may increase job stress by exacerbating deficits of social support in the workplace. Medium- and low-frequency WFH workers were more likely to have satisfactory job control; therefore, limiting WFH to three or fewer days per week may lead to better job stress management.

## Introduction

The coronavirus disease 2019 (COVID-19) caused by the severe acute respiratory syndrome coronavirus 2 (SARS-CoV-2), which began in December 2019, has repeatedly caused pandemics worldwide due to its viral mutations (Cascella et al., 2022; Dhawan et al., 2022). The Japanese government has repeatedly declared states of a quasi-emergency during the COVID-19 pandemic, and has strengthened infection prevention control measures, such as non-pharmaceutical interventions, e.g. wearing non-woven masks, physical distancing, indoor ventilation, and avoiding the 3 Cs (closed spaces, crowded places, and close-contact settings). In particular, the introduction of work from home (WFH) has proliferated rapidly to suppress population mobility and to avoid the three Cs in the workplace, and remains in place in many workplaces in 2022 (Persol Research and Consulting Co., 2022).

The International Labor Organization defines telework or remote work as ‘the work performed through the use of information communication technology (ICT’s such as smartphones, tablets, laptops and desktop computers) outside the employer’s premises’ (Messenger et al., 2017). The main forms of telework include ‘WFH,’ wherein employees work from their homes; ‘mobile work,’ wherein employees can work anywhere and anytime without depending on facilities; and ‘facility-based work,’ wherein employees work at facilities such as satellite offices, telework centers, and spot offices (Ministry of Internal Affairs and Communications, 2021). Telework is used in a broad sense, and includes all WFH, mobile work and facility-based work. In a Japanese survey on environmental forms of telework (duplicates due to multiple responses), 56/9% of teleworkers were involved in facility-based work, and 48.2% were involved in WFH. However, in 2020, during the COVID-19 pandemic, 90.3% were involved in WFH and 30.4% were involved in facility-based work (Ministry of Land, Infrastructure, Transport, and Tourism, 2021), and the number of workers who WFH has increased markedly.

Before the COVID-19 pandemic, research on telework or remote work in Japan focused on corporate management perspectives, such as cost reduction and business efficiency (Sato, 2019), and the research-based literature on its effects on work stress and mental health is limited. These studies have been conducted in several countries other than Japan. In a study of teleworkers in an American company, the degree of fatigue in teleworking was positively related to job demands (time pressure, role ambiguity, and role conflict) and negatively related to job resources (job autonomy, feedback, and job support), and indicated the need for support to ensure that those who telework are not isolated (Sardeshmukh et al., 2012). A study that used data from the 2010, 2012, and 2013 American Time Use Survey Well-Being Modules found that telework can increase psychological stress compared to working at the workplace (Younghwan Song, 2020), whereas another study found no significant relationship among teleworking, psychological stress, and wellbeing (Vander Elst et al., 2017), and there is no consistent finding of the relationship between telework and psychological stress.

Since the COVID-19 epidemic began, studies have reported on telework, particularly on the relationship between WFH, job stress, and mental health (Afonso et al., 2021; Giovanis & Ozdamar, 2021; Ipsen et al., 2021). A study on job stressors associated with WFH in 29 European countries conducted from mid-March to mid-May 2020 reported that the benefits of WFH might include ‘work-life balance, improved work efficiency, and better work control,’ whereas the disadvantages included ‘home office constraints, work uncertainties, and inadequate work tools’ (Ipsen et al., 2021). A previous study reported that those who WFH were less likely to answer that their financial situation will worsen in the future compared to those who do not WFH, while the mental health status of those who WFH worsened on evaluation using the General Health Questionnaire 12. The study suggested that workers eligible to WFH may be confident in their ability to secure work, but are exposed to the stresses of social isolation from coworkers, lack of contact with supervisors, and increased workloads (Giovanis & Ozdamar, 2021). A previous study of organizations and individuals with an interest in teleworking reported that WFH may negatively impact social exchange, job satisfaction, organizational commitment, and job performance (Kuruzovich et al., 2021). Thus, some evidence on WFH in the early phases of the COVID-19 epidemic suggests that WFH may increase workers’ job stress and adversely affect their mental health.

Since 2020, as a part of the Collaborative Online Research on the Novel-coronavirus and Work (CORoNaWork) Project, we have conducted studies on the physical and mental health impacts on Japanese workers during the COVID-19 epidemic. Using data from the CORoNaWork Project, we have evaluated the impact of WFH on the physical and mental health statuses among those who WFH in Japan. A cross-sectional analysis of the data from this project showed that high-frequency WFH (≥4 days per week) was associated with high job control and low subjective psychological stress in the early stages of the COVID-19 epidemic (Ikegami et al., 2022). However, those with medium-frequency WFH (WFH for 2–3 days per week) or low-frequency WFH (WFH between 1 d per week and 1 d per month) had higher work engagement compared to those who worked exclusively in the workplace (Nagata et al., 2021b). In these cross-sectional studies, WFH was suggested to be associated with good mental health among workers. However, there is a possibility that this phenomenon is transient, and further verification of the long-term mental health effects of WFH is required.

In Japan, WFH is becoming established as one of the work styles, but WFH might have a significant impact on the working environment. We believe it is important to continuously evaluate the impact of WFH on work stress. This study aimed to prospectively evaluate the impact of WFH on job stress among Japanese workers by using longitudinal data obtained through the CORoNaWork Project to ascertain strong evidence for the job stress impact of long-term WFH on workers during an infectious disease epidemic.

## Material and methods

### Study design and setting

This prospective cohort study was conducted at the University of Occupational and Environmental Health, Japan between December 2020 (baseline survey) and December 2021 (first follow-up survey). A portion of this survey was outsourced to Cross Marketing Inc. (Chiyoda-ku, Tokyo), which uses an online survey system to conduct self-administered questionnaire surveys. The study protocol for the CORoNaWork Project, using a sampling plan and participant recruitment procedures according to the Checklist for Reporting Results of Internet E-Surveys (CHERRIES), has been reported previously (Eysenbach, 2004; Fujino et al., 2021; Kawasumi et al., 2021).

### Participants

The target population of this study was Japanese workers aged 20–65 years at the time of the baseline survey. Sampling was stratified by gender, generation, residential area, and occupation, to reduce bias. Residential areas were divided into five groups of 47 prefectures according to the COVID-19 epidemic level. Occupations were divided into two groups: office and non-office workers. A total of 20 blocks were set up across five residential areas, two occupations, and two genders, with an equal number of participants sampled in each block. As we planned to survey 30,000 participants overall, we attempted to obtain at least 1,500 participants in each block.

Of the 4,700,000 survey monitors registered with the online survey systems of Cross Marketing, Inc., approximately 600,000 were e-mailed a request for their participation in this survey. Among them, 55,045 participated in the primary screening survey conducted by the contractor, and 33,087 met the eligibility criteria for participation in this survey.

**Figure 1.**
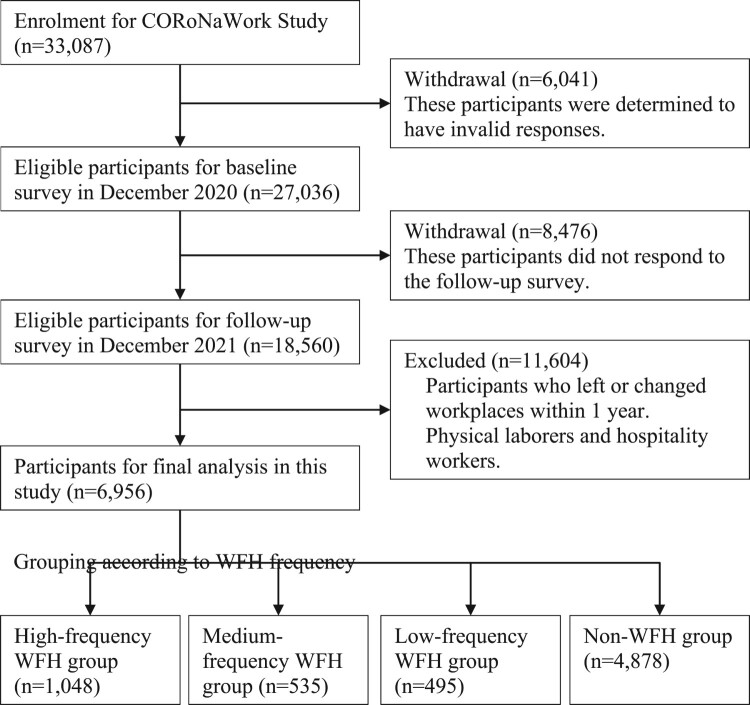
Flowchart depicting the selection of this study population.

Of the 33,087 initial participants, 27,036 were included in the CORoNaWork Project at baseline, after excluding participants who were determined to have fraudulent responses. The criteria used to determine fraudulent responses (i.e. exclusion criteria) were: those who had extremely short response time (≤6 min), extremely low weight (≤30 kg), extremely short (≤140 cm), contradictory responses among similar items throughout the questionnaire (e.g. contradictory responses to questions on marital status, the number of household members, and living situation), and incorrect responses to the questions designed to identify participants with fraudulent responses, (e.g. select the third largest number out of five) (Figure 1).

A follow-up survey was conducted in December 2021, 1 year after the baseline survey. In total, 18,560 participants were enrolled in the follow-up survey. Participants who changed their jobs or retired during the study period and those who were non-office workers (i.e. physical or hospital workers) were excluded from this study. Finally, 6,956 office workers (men: 3909; women: 3047) were included in the analysis (Figure 1).

The questionnaires used at the baseline and follow-up surveys were similar. However, some items were changed or deleted in the follow-up survey as they were difficult to handle as outcomes or owing to high cost of copyright royalties. Overall, the number of questions in the follow-up survey was fewer than in the baseline survey.

### Evaluation of job stress

**Figure 2.**
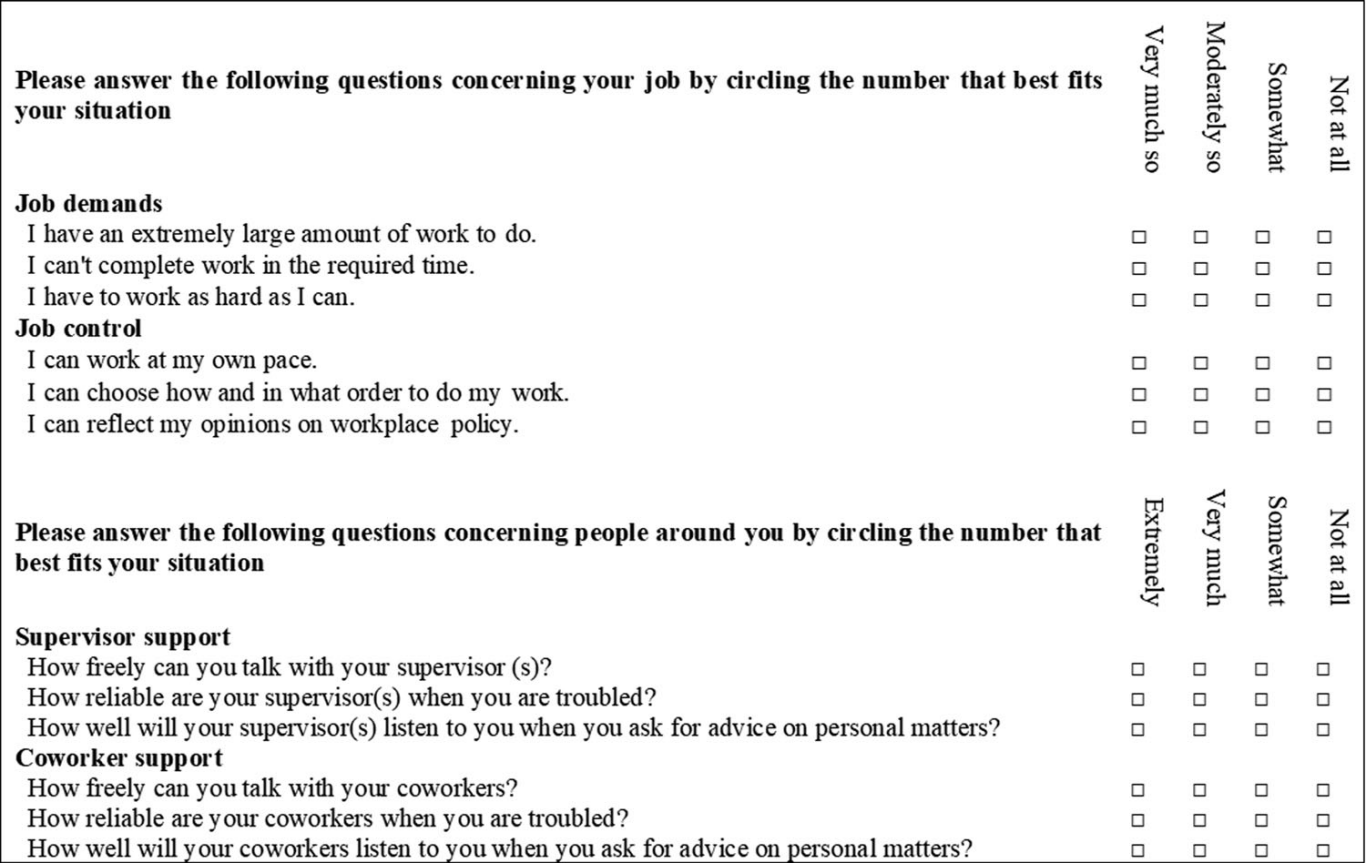
Question items of the Brief Job Stress Questionnaire (BJSQ) – 12 items excerpted version.

We used the Brief Job Stress Questionnaire (BJSQ) developed by Shimomitsu et al., a 57-item questionnaire for evaluating the degree of psychological job stress that is used in regular psychological stress examination conducted in accordance with the Japanese Occupational Health and Safety Law (Shimomitsu et al., 2000; Tsutsumi et al., 2018). In this study, 12 items measured the four subscales of job demand, job control, supervisor support, and coworker support (three items for each scale, on a 4-point Likert scale) (Figure 2). In the present analysis, we summed the item scores of the 4-point Likert scale to calculate the scores for the four job stressors. For each job stressor, the scores ranged from 3 to 12. In the present sample, the Cronbach’s alphas for job demands, job control, supervisor support, and coworker support were 0.88, 0.82, 0.88, and 0.90, respectively.

The cutoff value of ‘poor state’ was adopted for each subscale, referring to the evaluation criteria of the BJSQ (Shimomitsu et al., 2000). In this study, the scores for identifying poor job demands, poor job control, poor supervisor support, and poor coworker support were 3, 11–12, 11–12, and 10–12, respectively.

### Evaluation of WFH

We asked participants, ‘How often do you work from home? Please choose the one that most closely describes your current situation (4 days per week or more, 2–3 days per week, 1 day per week, less than 1 day per week [1–3 days per month], almost never).’ Participants were divided into four groups according to the WFH frequency: the high-frequency WFH group comprised participants working from home for 4 days or more per week; the medium-frequency WFH group included those working from home for 2–3 days per week; the low-frequency WFH group included those working from home for 1 day or less per week; and the non-WFH group comprised those who were not working from home.

### Outcomes and measurements

The scales of the BJSQ (job demands, job control, supervisor support, and coworker support) were used as outcome variables, and the WFH frequency group was used as the exposure variable.

The following variables were considered confounders: personal characteristics of gender, age (20–29, 30–39, 40–49, 50–59, and ≥60 years), and education (junior or senior high school, junior college or vocational school, or university or graduate school); familial factors, which included marital status (married or unmarried) and household size (1, 2, 3, or ≥4 people); and work-related factors, which included job type (regular employee, manager, or other) and workplace size (≤9, 10–49, 50–999, or ≥1000 employees).

These variables, with the exception of the workplace size, that were collected at baseline were used in the analysis conducted in this study. Moreover, the workplace size was ascertained at baseline; however, it was used in the subsequent analysis as more detailed information was collected at follow-up.

### Statistical analysis

To evaluate the impact of WFH frequency on the four subscales of the BJSQ, we used multilevel logistic regression analysis with two models in which the residential prefecture was nested as a random effect. We estimated the odds ratios (OR) for poor status for each subscale of the BJSQ. In the gender–age adjusted model, we treated gender, the age group, and the groups according to WFH frequency as fixed effects and the residential prefecture as a random effect. In the multivariate model, education, job type, workplace size, marital status, and the number of household members were added to the fixed effects in the gender–age adjusted model.

For all tests, the significance threshold was set at *P *< 0.05. Stata/SE Ver. 15.1 (StataCorp LLC, College Station, TX, USA) was used for the statistical analysis.

## Ethical considerations

This study was approved by the ethics committee of the University of Occupational and Environmental Health, Japan (Ref. Nos. R2-079 and R3-006). Online informed consent was obtained from all participants who were registered in the online survey system.

## Results

### Participants and descriptive data

Of the 6,956 participants whose data were analyzed, 2,078 (1,302 men and 776 women) had been working from home and 4,878 (2607 men and 2,271 women) had never worked from home. Table 1 shows the participants’ characteristics according to the WFH frequency at baseline. Those aged 50 years and older tended to more frequently WFH. In terms of work-related factors, those working in workplaces with ≤9 employees were more likely to be included in the group with the higher frequency of WFH. With regard to family-related factors, unmarried participants tended to more frequently WFH. Of the 1394 participants who lived alone (household members: one person), 276 participants (19.8%) were involved in high-frequency WFH. These proportions decreased as the number of family members increased (two people: 17.1%, three people: 14.1%, four people or more: 10.6%).

**Table 1.** Characteristics by four groups according to the WFH frequency at baseline.

Variables	WFH frequency							
	High (*n* = 1,048)		Medium (*n* = 535)		Low (*n* = 495)		Non-WFH (*n* = 4,878)	
	*n*	(%)	*n*	(%)	*n*	(%)	*n*	(%)
Gender, men	637	(60.8)	320	(59.8)	345	(69.7)	2607	(53.4)
Age (years)								
20–29	30	(2.9)	22	(4.1)	25	(5.1)	183	(3.8)
30–39	126	(12)	74	(13.8)	59	(11.9)	724	(14.8)
40–49	297	(28.3)	150	(28)	142	(28.7)	1642	(33.7)
50–59	431	(41.1)	227	(42.4)	204	(41.2)	1856	(38)
60–69	164	(15.6)	62	(11.6)	65	(13.1)	473	(9.7)
Educational background								
Junior or senior high school	195	(18.6)	43	(8)	70	(14.1)	1236	(25.3)
Junior college or vocational school	208	(19.8)	79	(14.8)	65	(13.1)	976	(20)
University or graduate school	645	(61.5)	413	(77.2)	360	(72.7)	2666	(54.7)
Workplace size (no. of employees)^†^								
≤9	582	(55.5)	89	(16.6)	84	(17)	1100	(22.6)
10–49	80	(7.6)	99	(18.5)	98	(19.8)	1474	(30.2)
50–999	220	(21)	232	(43.4)	205	(41.4)	1817	(37.2)
≥1000	166	(15.8)	115	(21.5)	108	(21.8)	487	(10)
Job type								
Regular employee	314	(30)	272	(50.8)	216	(43.6)	2446	(50.1)
Manager	99	(9.4)	124	(23.2)	128	(25.9)	669	(13.7)
Others	635	(60.6)	139	(26)	151	(30.5)	1763	(36.1)
Marital status, unmarried	552	(52.7)	208	(38.9)	173	(34.9)	1998	(41)
No. of household members (persons)								
1	276	(26.3)	107	(20)	90	(18.2)	921	(18.9)
2	311	(29.7)	134	(25)	110	(22.2)	1260	(25.8)
3	256	(24.4)	154	(28.8)	149	(30.1)	1260	(25.8)
≥4	205	(19.6)	140	(26.2)	146	(29.5)	1437	(29.5)

WFH: work from home.

^†^ Although data on the size of the company where the participants have worked had been collected in the baseline survey, data on the size of the workplace was collected in the follow-up survey to obtain more detailed information.

Table 2 shows the results of each BJSQ subscale of the participants by the WFH frequency at follow-up. The medium-frequency WFH group tended to have a lower proportion of poor job control, whereas the high-frequency WFH group and the non-WFH group tended to have a higher proportion of poor job control. The high-frequency WFH group tended to have a higher proportion of poor supervisor support and poor coworker support.

**Table 2.** The results of each BJSQ subscales by groups according to WFH frequency at follow-up.

BJSQ subscales Evaluation	WFH frequency							
	High (*n* = 1,048)		Medium (*n* = 535)		Low (*n* = 495)		Non-WFH (*n* = 4,878)	
	*n*	(%)	*n*	(%)	*n*	(%)	*n*	(%)
Job demands								
Fair (score: 4–12)	974	(92.9)	502	(93.8)	457	(92.3)	4541	(93.1)
Poor (score: 3)	74	(7.1)	33	(6.2)	38	(7.7)	337	(6.9)
Job control								
Fair (score: 3–10)	1003	(95.7)	520	(97.2)	478	(96.6)	4595	(94.2)
Poor (score: 11–12)	45	(4.3)	15	(2.8)	17	(3.4)	283	(5.8)
Supervisor support								
Fair (score: 3–10)	664	(63.4)	470	(87.9)	440	(88.9)	4157	(85.2)
Poor (score: 11–12)	384	(36.6)	65	(12.1)	55	(11.1)	721	(14.8)
Coworker support								
Fair (score: 3–9)	641	(61.2)	451	(84.3)	442	(89.3)	4070	(83.4)
Poor (score: 10–12)	407	(38.8)	84	(15.7)	53	(10.7)	808	(16.6)

BJSQ: Brief Job Stress Questionnaire, WFH: work from home.

### Comparison of job stress among the four groups according to WFH frequency

Table 3 shows the ORs for poor status of each BJSQ subscale. In the age-adjusted model, compared to the non-WFH group, the high, medium, and low WFH groups were likely to have similar levels of poor job demands (OR = 1.08; 95% CI = [0.83–1.41], OR = 0.91; 95% CI = [0.62–1.32], and OR = 1.13; 95% CI = [0.79–1.61], respectively). These results are similar to those of the multivariate model.

**Table 3.** Comparison of the subscales of the BJSQ among four groups according to WFH frequency.

BJSQ subscales	WFH frequency	Gender–age adjusted			Multivariate		
		OR	[95% CI]	*P*	OR	[95% CI]	*P*
Job demands	High	1.08	[0.83–1.41]	0.561	1.21	[0.91–1.61]	0.189
	Medium	0.91	[0.62–1.32]	0.613	0.82	[0.56–1.21]	0.316
	Low	1.13	[0.79–1.61]	0.505	1.03	[0.72–1.48]	0.869
	Non	Ref.			Ref.		
Job control	High	0.77	[0.55–1.06]	0.105	0.77	[0.55–1.09]	0.137
	Medium	0.48	[0.28–0.81]	0.006	0.45	[0.26–0.77]	0.003
	Low	0.60	[0.36–0.99]	0.047	0.58	[0.35–0.96]	0.035
	Non	Ref.			Ref.		
Supervisor support	High	3.35	[2.88–3.90]	<0.001	2.39	[2.02–2.82]	<0.001
	Medium	0.81	[0.61–1.06]	0.125	0.93	[0.70–1.24]	0.629
	Low	0.73	[0.54–0.98]	0.036	0.83	[0.61–1.12]	0.213
	Non	Ref.			Ref.		
Coworker support	High	3.26	[2.81–3.79]	<0.001	2.29	[1.95–2.70]	<0.001
	Medium	0.96	[0.75–1.22]	0.718	1.09	[0.85–1.41]	0.494
	Low	0.62	[0.46–0.84]	0.002	0.69	[0.51–0.93]	0.016
	Non	Ref.			Ref.		

BJSQ: Brief Job Stress Questionnaire, WFH: work from home, CI; confidence interval.

In the age-adjusted models, compared to the non-WFH group, the medium and low WFH groups were less likely to have poor job control (OR = 0.48; 95% CI = [0.28–0.81] and OR = 0.60, 95% CI = [0.36–0.99], respectively). These results are similar to those of the multivariate model. (OR = 0.45; 95% CI = [0.26–0.77] and OR = 0.58, 95% CI = [0.35–0.96], respectively).

Compared to the non-WFH group, the high WFH group was more likely to have poor supervisor support (OR = 3.35; 95% CI = [2.88–3.90], OR = 2.39; 95% CI = [2.02–2.82], respectively). Similarly, in both models, compared to the non-WFH group, the high WFH group was more likely to have poor coworker support (OR = 3.26; 95% CI = [2.81–3.79], OR = 2.29; 95% CI = [1.95–2.70], respectively).

## Discussion

This study prospectively evaluated the impact of WFH on job stress, as measured by the BJSQ, during the COVID-19 pandemic among Japanese workers. We examined the impact of WFH frequency on job demands. In our cross-sectional baseline study, the Job Contents Questionnaire (JCQ) was used to quantify parameters regarding job stress, and we treated the JCQ subscale scores as quantitative variables. We found that workers with higher frequency WFH tended to have significantly lower job demands (Ikegami et al., 2022). In this study, we found no significant differences in the impact of WFH frequency on job demands. There may be differences in the trends of WFH frequency and job demands between this study and our previous cross-sectional study, even though direct comparisons are not possible due to the different questionnaires used in these two studies. The baseline survey was conducted less than 1 year after WFH was instituted in Japan as a pandemic countermeasure for COVID-19. WFH was mainly instituted during this period as one of the COVID-19 prevention measures considering social conditions, rather than as an aspect of business continuity plans for companies. Therefore, the job demands required of WFH workers may have inevitably decreased. However, the reason for a loss of significant difference in job demands according to the frequency of WHF at the time of the follow-up survey may be because corporate labor management and operational controls for workers with WFH became functional during this period. As studies before the COVID-19 epidemic reported that telework may tend to lengthen working hours and increase job demands (Noonan & Glass, 2012), further investigation is needed to clarify these speculations.

With regard to the impact of WFH frequency on job control, we reported that workers with a higher frequency of WFH had more job control at baseline in our cross-sectional study (Ikegami et al., 2022). In the present study, we found that poor job control was significantly lower among workers with medium- and low-frequency WHF compared to workers without WFH; however, there was no significant difference between the workers with high-frequency WFH and the non-WFH workers. Therefore, we infer that makeshift WFH was undertaken during the early phase of the COVID-19 epidemic, and workers with WFH had fewer work instructions and more freedom in their work. Alternatively, long-term WFH could reveal the limitations of the work environment and work tools (such as workroom, desk/chair, internet environment, and personal computer peripheral device) and the difficulties in managing work progress under WFH (Ipsen et al., 2021; Matsugaki et al., 2021; Nagata, et al., 2021a). Furthermore, the frequency of telework may factor into the difficulty of managing work progress. In this study, we found that high-frequency WFH would not lead to good job control.

Furthermore, the results of the impact of WFH frequency on social support in the workplace (i.e. supervisor and coworker support) showed that workers with high-frequency WFH had significantly high ORs of poor supervisor support and poor coworker support compared to the standard reference – non-WFH workers. These results are consistent with those of our baseline cross-sectional study, which reported that workers with high-frequency WFH were associated with lower supervisor and coworker support, even after adjusting for gender–age and work-related factors. In contrast, the results of our previous study differ from that of the present study in that the significant difference between workers with high-frequency WFH and those without WFH disappeared after additionally adjusting for familial factors (marital status and family living together) (Ikegami et al., 2022). In this study, the ORs of poor social support of workers with high-frequency WFH remained significantly high after multivariate adjustment, including for familial factors. Several international studies also report that WFH may have decreased social interaction and organizational commitment (Giovanis & Ozdamar, 2021; Kuruzovich et al., 2021), and the findings of the negative impact of WFH on social support are consistent. In this longitudinal study, we found that continuous high-frequency WFH is a direct cause of lower social support. Quality communication and peer support within organizations positively affect mental health and enhance social capital and organizational resilience during contagious epidemics (Pollock et al., 2020). Workplaces may need to strengthen their online communication systems and information and communication technology literacy training when teleworking.

Studies have reported the effects of teleworking on workers’ job stressors and mental health, albeit with inconsistent findings; increasing or decreasing job stress has been reported depending on various confounding factors and moderator adjustments (Anderson et al., 2015; Bentley et al., 2016; Gajendran & Harrison, 2007; Grant et al., 2013; Sardeshmukh et al., 2012; Vander Elst et al., 2017). Nijp et al. (2016) reported that teleworking would not affect major psychosocial job stress, such as the degree of work hours, job demands, job control, and social support. The results of the present study suggest that job demands and control are comparable between workers with high-frequency WFH and those without WFH; however, those with high-frequency WFH have less supervisor and coworker support than workers without WFH. Those with high-frequency WFH may experience increased job stress due to low social support. Whereas workers with occasional WFH may experience reduced job stress since they have better job control compared to those without WFH. The risk of mental health deterioration due to WFH depends on the degree of teleworking frequency, and a significantly lower prevalence of depression has been reported in those who telework for less than 8 h per month compared with workers who did not telework (Henke et al., 2016).

We believe that it may be necessary to establish an occupational health support system, such as an advisory system for teleworkers, to strengthen communication among teleworkers, with mental health education for supervisors to help manage teleworkers’ mental health. Particularly, considering the frequency of telework, we suggest that limiting WFH to three or fewer days per week and interweaving it with work at the workplace may lead to better job stress management. Job stress associated with telework, including WFH, may be a future issue for occupational mental health. Even when the COVID-19 pandemic is under control, it will be necessary to continue to investigate changes in the form and frequency of teleworking in Japanese workers and the effects of telework on job stress, according to the teleworkers.

### Limitations

This study had several limitations. First, this study only included participants who were residing in Japan and were registered in the online survey system of Cross Marketing, Inc. Therefore, the study sample may not be representative of general workers, and the generalizability of the study’s results may necessitate caution. For example, many participants could have high computer skills and ICT literacy as this study was conducted using the internet. Thus, this factor may have affected the study results. In addition, because teleworking is implemented according to Japanese labor-related laws and regulations or various Japanese business guidelines, the WFH scenario evaluated in Japan may be different from the actual teleworking situation in other countries. Next, we did not consider the changes in WFH frequency among participants. The baseline survey for this study was conducted in December 2020, when the third wave of the COVID-19 pandemic was expanding nationwide, and a high-frequency WFH may have occurred at baseline. The WFH frequency would have constantly fluctuated as the COVID-19 pandemic grew and may have subsided repeatedly even within a single year, as a state of actual or quasi-emergency was declared by the government or as alerts regarding the COVID-19 epidemic by each prefecture were announced each time. Therefore, it is difficult to analyze additional variables, such as the amount of change in participants’ WFH frequency. However, we believe that the policies and procedures concerning WFH for each workplace could be identified and established in the early-stage COVID-19 epidemic. Lastly, because this study has evaluated WFH during the COVID-19 pandemic, there may be some differences from the effects of WFH on job stress during the normal stable periods. However, we believe that this study was able to ensure a certain degree of rationality by adjusting for potential confounding factors, in addition to the longitudinal analysis.

## Conclusion

In this study, we prospectively evaluated the impact of WFH on job stress among Japanese workers, using longitudinal data obtained from the CORoNaWork Project. We found that workers with high-frequency WFH experience adverse effects in terms of coworker and supervisor support among the four subjective job stresses, including job demands, job control, supervisor support, and coworker support.

The workers with medium- and low-frequency WHF were more likely to have better job control compared to workers without WFH; however, no significant effect was observed in workers with high-frequency WFH, who may have high job stress. We suggest that limiting WFH to three or fewer days per week and interweaving it with work at the workplace may lead to better job stress management.

## Data Availability

The data produced in the Collaborative Online Research on the Novel-coronavirus and Work (CORoNaWork) Project are available upon reasonable request to the corresponding authors.
